# The URX oxygen-sensing neurons in *C. elegan*s are ciliated

**DOI:** 10.17912/micropub.biology.000303

**Published:** 2020-09-20

**Authors:** Anna Kazatskaya, Lisa Yuan, Niko Amin-Wetzel, Alison Philbrook, Mario de Bono, Piali Sengupta

**Affiliations:** 1 Brandeis University, Waltham, MA 02454; 2 Institute of Science and Technology Austria (IST Austria), Klosterneuburg, Austria

**Figure 1. The URX neurons contain a cilium-like structure at their distal dendritic ends:**
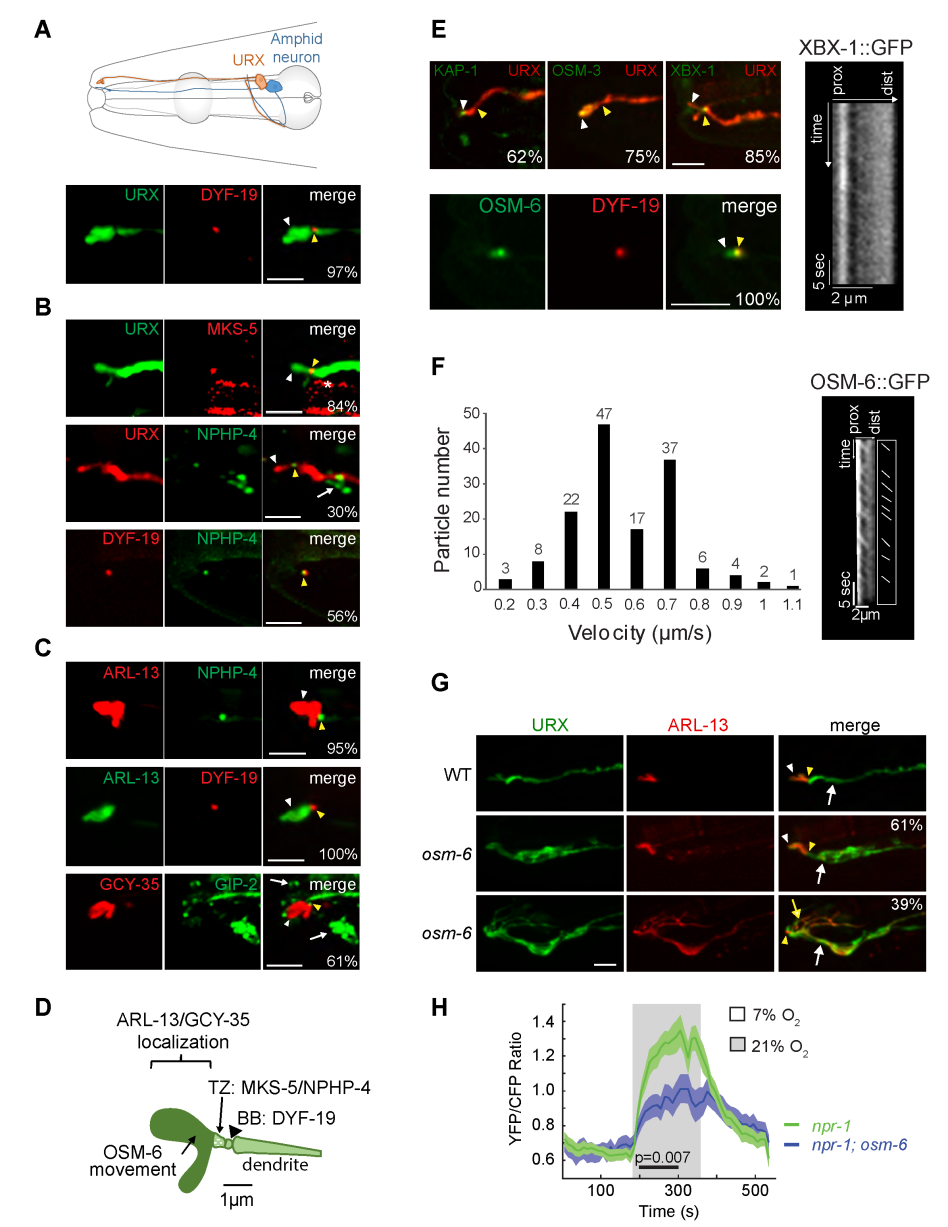
**A)** (Top) Schematic of an URX (orange) and representative amphid sensory neuron (blue) in the head of an adult *C. elegans* hermaphrodite. Only neurons on the left are shown. (Bottom) Localization of the basal body-associated protein DYF-19::tagRFP expressed under the *gcy-36* promoter at the URX distal dendritic ends. URX is marked via expression of *gcy-32*p::GFP. **B)** (Top two rows) Localization of the transition zone proteins MKS-5::tagRFP and NPHP-4::GFP in URX. MKS-5::tagRFP and NPHP-4::GFP were expressed under the *gcy-32* and endogenous promoters, respectively. Arrow indicates localization of NPHP-4 in other cilia, asterisk indicates background fluorescence in the pharynx. URX is marked with *gcy-32*p::GFP or *gcy-36*p::RFP. (Bottom row) Colocalization of DYF-19::tagRFP and NPHP-4::GFP at the URX distal dendritic ends. **C)** (Top two rows) Colocalization of ARL-13::tagRFP and ARL-13::GFP with NPHP-4::GFP and DYF-19::tagRFP, respectively, at the URX distal dendritic ends. ARL-13 was expressed under the *gcy-32* promoter, NPHP-4 and DYF-19 were expressed under the *gcy-36* promoter. (Bottom row) Colocalization of GCY-35::mKate with GIP-2::GFP at the URX distal dendritic ends. GCY-35::mKate was expressed under the *gcy-36* promoter; GIP-2::GFP was expressed from its endogenous locus (Harterink *et al.* 2018); arrows indicate localization in other cells. **D)** Summary of localization patterns of examined fusion proteins at the URX sensory endings. TZ: transition zone, BB: basal body. **E)** Localization of KAP-1::GFP, OSM-3::GFP, XBX-1::GFP, OSM-6::GFP and DYF-19::tagRFP fusion proteins at the URX distal dendritic ends. Fusion proteins were expressed under the *gcy-32* (OSM-3, KAP-1, OSM-6) or *gcy-36* (XBX-1, DYF-19) promoters. URX was visualized via expression of *gcy-36*p::RFP. (Right) Representative kymograph of XBX-1::GFP movement in the URX sensory endings. **F)** (Left) Histogram of OSM-6::GFP anterograde velocity in the URX cilium-like structure. OSM-6::GFP was expressed in URX under the *gcy-32* promoter. (Right) Representative kymograph and schematic of OSM-6::GFP movement in the URX cilium-like structure. n= total of 148 particles from 20 animals. **G)** Localization of ARL-13::tagRFP in URX (marked via expression of *gcy-32*p::GFP) in wild-type and *osm-6(p811)* animals. White and yellow arrows indicate the main dendrite and dendritic branches, respectively. **H)** Average YFP/CFP ratio change (solid lines) in URX neurons expressing YC2.60 in animals of the indicated genotypes in response to a 7%-21% oxygen shift. Shaded areas are SEM. The black bar indicates the time intervals used for statistical comparisons (Mann-Whitney U Test). Only responding neurons were included for generating the graph and the statistical analysis (WT: 21/21; *osm-6*: 20/44). Since the laboratory N2 strain contains a gain-of-function variant of the NPR-1 neuropeptide Y-like receptor that inhibits some oxygen responses (Weber *et al.* 2010; Busch *et al.* 2012), imaging experiments were performed in an *npr-1(ad609)* loss-of-function background. In all relevant panels, white and yellow arrowheads indicate the presumptive cilia-like structure and the basal body/transition zone, respectively. Anterior is at left in all image panels. Numbers in lower right corners indicate the percentage of animals exhibiting the observed localization pattern; n > 21 for each. Scale bars: 5 µm unless otherwise noted.

## Description

A subset of sensory neurons in *C. elegans* contains compartmentalized sensory structures termed cilia at their distal dendritic ends (Ward *et al.* 1975; Perkins *et al.* 1986; Doroquez *et al.* 2014). Cilia present on different sensory neuron types are specialized both in morphology and function, and are generated and maintained via shared and cell-specific molecules and mechanisms (Perkins *et al.* 1986; Evans *et al.* 2006; Mukhopadhyay *et al.* 2007; Mukhopadhyay *et al.* 2008; Morsci and Barr 2011; Doroquez *et al.* 2014; Silva *et al.* 2017). The bilaterally symmetric pair of URX oxygen-sensing neurons in the *C. elegans* head (Figure 1A) is thought to be non-ciliated (Ward *et al.* 1975; Doroquez *et al.* 2014) but nevertheless exhibits intriguing morphological similarities with ciliated sensory neurons. URX dendrites extend to the nose where they terminate in large bulb-like complex structures (Ward *et al.* 1975; Doroquez *et al.* 2014; Cebul *et al.* 2020) (Figure 1A). These structures concentrate oxygen-sensing signaling molecules (Gross *et al.* 2014; Mclachlan *et al.* 2018) suggesting that similar to cilia, these structures are specialized for sensory functions. Microtubule growth events similar to those observed in ciliated sensory neurons were also reported at the distal dendritic regions of URX, implying the presence of a microtubule organizer such as a remodeled basal body (Harterink *et al.* 2018). Moreover, a subset of ciliary genes is expressed in URX (Kunitomo *et al.* 2005; Harterink *et al.* 2018; Mclachlan *et al.* 2018). We tested the hypothesis that URX dendrites contain cilia at their distal ends.

We first examined the localization of proteins associated with different ciliary compartments in URX. The basal body component DYF-19/FBF1 (Wei *et al.* 2013) was enriched at single puncta at the distal dendritic ends of URX (Figure 1A). A similar localization pattern was previously reported for the centrosome-associated protein gamma-tubulin (Harterink *et al.* 2018). The MKS-5::tagRFP (Williams *et al.* 2011) and NPHP-4::GFP (Jauregui and Barr 2005; Williams *et al.* 2011) transition zone fusion proteins were also localized to single puncta at the URX dendritic tips, distal to the region of DYF-19 localization (Figure 1B). In addition, ARL-13::GFP, a well-characterized ciliary membrane marker (Cevik *et al.* 2010; Li *et al.* 2010) was restricted to a region distal to the DYF-19::tagRFP and NPHP-4::GFP puncta (Figure 1C). The mKate-tagged oxygen-sensing soluble guanylyl cyclase GCY-35 was also highly enriched at the distal dendritic ends of URX in a domain that was defined proximally by the GIP-2 component of gamma-TuRC expressed from its endogenous locus (Figure 1C) (Wang *et al.* 2015; Harterink *et al.* 2018). We noted that overexpression of ARL-13 and GCY-35 resulted in an expansion of the presumptive cilium-like structure in URX; overexpression of ARL-13 has previously been reported to alter cilia morphology in other cell types (Hori *et al.* 2008; Larkins *et al.* 2011). Together, these observations imply the presence of a cilium-like structure at the distal dendritic ends of URX that houses signaling molecules, and that is delineated proximally by a transition zone and basal body (summarized in Figure 1D). We speculate that the short length of this structure may have precluded its identification in previous ultrastructural studies (Ward *et al.* 1975; Perkins *et al.* 1986; Doroquez *et al.* 2014).

We next investigated whether intraflagellar transport (IFT) necessary for cilia generation and maintenance (Rosenbaum and Wittman, 2014) could be detected in this cilium-like organelle in URX. Although the KAP-1 kinesin-2 motor component, OSM-3 homodimeric motor, and XBX-1 dynein light chain subunit were localized to the very distal ends of the URX dendrites, we were unable to detect movement of these proteins under standard conditions (Figure 1E). Similar to the localization of these proteins, the IFT core component OSM-6::GFP was also present in a small domain distal to that occupied by the DYF-19::tagRFP puncta (Figure 1E). However, we observed anterograde movement of OSM-6::GFP in the URX dendritic ends at an average speed of 0.61 ± 0.20 µm/sec (Figure 1F), consistent with kinesin-2-mediated transport. We were unable to detect and/or quantify retrograde movement of OSM-6::GFP. Unlike in other characterized sensory cilia which typically exhibit robust IFT, episodes of OSM-6::GFP anterograde movement in the presumptive URX cilia-like structure were infrequent and could be observed in only ~20% of examined neurons under specific conditions (see Methods).

We asked whether IFT proteins are necessary for the structure and/or function of the URX cilium-like structure. Although we were unable to definitively determine whether the very short cilium-like structure was further truncated in *osm-6* mutants, we found that ARL-13::tagRFP was mislocalized to the URX dendrite in all examined *osm-6* mutants (Figure 1G). Interestingly, ~40% of URX neurons in *osm-6* mutants also exhibited multiple branches emanating from their distal dendritic ends; these branches contained ARL-13::GFP (Figure 1G). Similar, albeit less extensive, branches (also referred to as ‘posterior projections’) have been reported from the distal ends of a subset of ciliated sensory neuron dendrites in animals mutant for IFT genes in *C. elegans* (Lewis and Hodgkin 1977; Perkins *et al.* 1986; Fujiwara *et al.* 1999; Murayama *et al.* 2005; Kunitomo and Iino 2008; Maurya *et al.* 2019).

URX responds to a 7%-21% rise in oxygen with a tonic increase in intracellular calcium levels (Zimmer *et al.* 2009; Busch *et al.* 2012). We found that only 45% (20/44) of URX neurons in *osm-6(p811)* mutants exhibited oxygen-evoked responses as assessed via changes in YC2.60 fluorescence, as compared to 100% of neurons (21/21) in wild-type animals. Moreover, response amplitudes were significantly decreased in responding URX neurons in *osm-6* mutants (Figure 1H). We conclude that a subset of IFT molecules is necessary for the morphological and functional integrity of the cilia-like compartment in URX.

The sensory compartments at the distal ends of URX dendrites exhibit several features characteristic of cilia. Basal body, transition zone, IFT, and ciliary membrane and membrane-associated markers, including sensory signaling molecules, are localized in domains whose relative organization at the URX dendritic ends is similar to those present in *bona fide* sensory cilia. Moreover, OSM-6 appears to undergo IFT in a subset of URX cilia-like structures and is required for correct neuronal morphology, ciliary protein localization and sensory functions. However, we did not observe movement of any additional IFT proteins including motors. It is possible that motor movement in the short URX cilium is difficult to detect due to technical considerations. Alternatively, URX may employ cell-specific mechanisms including cell-specific motors to build its cilia. Finally, environmental manipulations have been shown to alter neuronal and cilia morphology including URX dendritic morphology in *C. elegans* (Albert and Riddle 1983; Mukhopadhyay *et al.* 2008; Procko *et al.* 2011; Schroeder *et al.* 2013; Cohn *et al.* 2019). Thus, trafficking in URX cilia may be modulated in a context-specific manner, perhaps to regulate cilium length or protein composition in response to specific cues. In the future, it will be interesting to correlate IFT in URX cilia with neuronal responses under defined external and internal conditions.

## Methods

***C. elegans* growth**

*C. elegans* strains were maintained at 20°C under normoxic conditions on standard NGM agar plates seeded with *E. coli* OP50 unless specified otherwise (all strains used in this work are indicated in Table 1). Mutant and transgenic strains were generated using standard methods. The presence of the desired mutation was confirmed by sequencing. Plasmids were injected at 1-10 ng/µl (see Table 2) with co-injection markers (*unc-122*p*::gfp*, *unc-122*p*::rfp* or *unc-122*p*::dsRed*) injected at 30 ng/µl.

**Molecular biology**

All DNA constructs were generated using standard cloning techniques, subcloned into the pPD95.77 *C. elegans* expression plasmid (A.Fire, Stanford University) and validated by restriction enzyme digestion and sequencing. Plasmids used in this work are indicated in Table 2.

**Microscopy**

One day-old adults were anesthetized using 10mM tetramisole (Sigma), diluted in M9 buffer and mounted onto 10% agarose pads. Live anesthetized animals were imaged on an inverted spinning disk microscope (Zeiss Axio Observer with a Yokogawa CSU-22 spinning disk confocal head). Images of protein localization were generated by collecting optical sections (*z*-stack) every 0.2-0.27 µm using Plan Apochromat 100x/1.40 NA or 63X/1.20 NA oil immersion objectives. Maximum intensity projection (*z*-stacks) images were generated using SlideBook 6.0 software (Intelligent Imaging Innovations, 3i). Enhancement of brightness and contrast across the entire image, as well as image rotations were performed using ImageJ/Fiji (NIH) software. Each strain was imaged independently on at least two different days. Two or more transgenic lines were generated and examined for each transgenic strain.

**IFT**

L4 animals were picked 18-24 hours prior to imaging and maintained at 15°C on unseeded NGM plates under normoxic conditions. OSM-6 IFT movement was observed only under these conditions in very young adults. 2D timelapse videos of OSM-6 and XBX-1 were recorded using SlideBook 6.0 software (Intelligent Imaging Innovations, 3i) using an inverted spinning disk confocal microscope (Zeiss Axio Observer with a Yokogawa CSU-22 spinning disk confocal head) using a Plan Apochromat 63X NA 1.2 oil immersion objective. The kymographs were generated using the MultipleKymographs plugin in ImageJ/Fiji (NIH). Individual particle tracks were manually traced.

**Calcium imaging**

Imaging of the calcium response in URX neurons was carried out across 3 days in animals expressing *gcy-37*p::YC2.60 as previously described (Chen *et al.* 2017). Briefly, movies were recorded with a Nikon AZ100 microscope equipped with a Nikon ×2 AZ-Plan Fluor objective and Hamamatsu ORCA-FLASH4.0 cameras using NIS-Elements software and 500ms exposure time. Excitation light produced by a Nikon Intensilight C-HGFI was passed through a 438/24nm filter and a Semrock FF458DiO2 dichroic. The emission light was split using a Cairn Research TwinCam dual camera adapter and passed through CFP and YFP filters (483/32nm and 542/27nm respectively), and a DC/T510LPXRXTUf2 dichroic. Dermabond adhesive was used to immobilize young adults on agar pads while keeping the nose exposed. 1ml of concentrated OP50 in M9 buffer was applied to the head and allowed to dry before the slide was transferred to the imaging chamber. In the imaging chamber, animals were exposed to 7% oxygen for 2 minutes before starting the experiment in which 7% then 21% then 7% oxygen concentrations were sequentially flowed into the imaging chamber for 3 min intervals. The recorded data was processed using Neuron Analyzer, a custom-written Matlab program.

## Reagents

**Table 1.** List of strains used in this work.

**Table:** 

**Strain**	**Genotype**	**Source**
PY11327	*iaIs25 [gcy-37*p*::gfp, unc-119(+)]; oyEx643 [gcy-36*p*::dyf-19::tagRfp, unc-122*p*::mCherry]*	Inna Nechipurenko
PY10377-79	Ex*[gcy-32*p*::gfp, gcy-32*p*::mks-5::tagRfp; unc-122*p*::Rfp]* Lines 2A, 6A, 21A	This work
PY10380	Ex*[nphp-4*p*::nphp-4::gfp*, *gcy-36*p*::tagRfp*, *unc-122*p*::Rfp]* Line 2A	This work
PY10381	Ex*[gcy-32*p*::osm-3b::gfp, gcy-36*p*::tagRfp, unc-122*p*::Rfp]* Line 10B	This work
PY10382-84	Ex*[gcy-32*p*::kap-1::gfp, gcy-36*p*::tagRfp, unc-122*p*::Rfp]* Lines 7E, 11B, 16C	This work
PY11316	*iaIs25 [gcy-37*p*::gfp, unc-119(+)]; oyEx645 [gcy-32*p*::arl-13::tagRfp, unc-122*p*::mCherry]*	Inna Nechipurenko
PY10386	*osm-6(p811); iaIs25 [gcy-37*p*::gfp];* Ex*[gcy-32*p*::arl-13::tagRfp, unc-122*p*::dsRed]*	This work
PY10357	Ex*[gcy-32*p*::osm-6::gfp, gcy-36*p*::tagRfp, unc-122*p*::Rfp]*	This work
PY10388-90	Ex*[gcy-36*p*::xbx-1::gfp*, *gcy-36*p*::tagRfp*, *unc-122*p*::Rfp]* Lines 13C, 13A, 4A	This work
PY10391-94	Ex*[gcy-32*p*::arl-13::tagRfp, gcy-36*p*::nphp-4::gfp, unc-122*p*::gfp]* Lines 1-4	This work
PY10395	Ex*[gcy-36*p*::nphp-4::gfp, gcy-36*p*::dyf-19::tagRfp, unc-122*p*::gfp]* Line 8	This work
PY10396-99	Ex*[gcy-36*p*::dyf-19::tagRfp; gcy-32*p*::osm-6::gfp, unc-122*p*::Rfp]* Lines 2, 4, 5A, 5B	This work
PY10310	*gip-2(lt19[gip-2::gfp::loxP::cb-unc-119(+)::loxP]); hrtSi57[gcy-36*p*::gcy-35::mKate-intra]*	Martin Harterink
PY10387	Ex*[gcy-36*p*::dyf-19::tagRfp, gcy32*p*::arl-13::gfp, unc-122*p*::Rfp]* Line 2	This work
PY10352	*osm-6(p811); npr-1 (ad609),* Ex*[gcy-37*p*::YC2.60]*	This work

**Table 2.** List of plasmids used in this work.

**Table:** 

**Plasmid**	**Description**	**Source** (injection concentration)
PSAB1231	*gcy-32*p*::mks-5cDNA::tagRfp*	Inna Nechipurenko (5 ng/µl)
PSAB1224	*gcy-36*p*::tagRfp*	This work (5 ng/µl)
PSAB1028	*nphp-4*p*::nphp-4::gfp*	Maureen Barr (10 ng/µl)
PSAB1225	*gcy-32*p*::osm-3b::gfp*	This work (10 ng/µl)
PSAB1226	*gcy-32*p*::kap-1::gfp*	This work (10 ng/µl)
PSAB1215	*gcy-32*p*::arl-13::tagRfp*	Inna Nechipurenko (10 ng/µl)
PSAB1227	*gcy-32*p*::osm-6::gfp*	This work (10 ng/µl)
PSAB1228	*gcy-36*p*::xbx-1::gfp*	This work (10 ng/µl)
PSAB1229	*gcy-36*p*::nphp-4::gfp*	This work (10 ng/µl or 1ng/µl)
PSAB1216	*gcy-36*p*::dyf-19::tagRfp*	Inna Nechipurenko (1 ng/µl or 5 ng/µl)
PSAB1230	*gcy-32*p*::arl-13::gfp*	This work (10 ng/µl)
